# Protective Effects of Myricetin on Benzo[a]pyrene-Induced 8-Hydroxy-2′-Deoxyguanosine and BPDE-DNA Adduct

**DOI:** 10.3390/antiox9050446

**Published:** 2020-05-21

**Authors:** Seung-Cheol Jee, Min Kim, Kyeong Seok Kim, Hyung-Sik Kim, Jung-Suk Sung

**Affiliations:** 1 Department of Life Science, Dongguk University-Seoul, Biomedi Campus, 32 Dongguk-ro, Ilsandong-gu, Goyang-si, Gyeonggi-do 10326, Korea; markjee@naver.com (S.-C.J.); pipikimmin@naver.com (M.K.); 2 Department of Division of Toxicology, School of Pharmacy, Sungkyunkwan University-Suwon, Gyeonggi-do 16419, Korea; caion123@nate.com (K.S.K.); hkims@skku.edu (H.-S.K.)

**Keywords:** Benzo[a]pyrene, myricetin, oxidative stress, BPDE-DNA adduct, phase detoxifying enzyme

## Abstract

Benzo[a]pyrene (B[a]P), a group 1 carcinogen, induces mutagenic DNA adducts. Myricetin is present in many natural foods with diverse biological activities, such as anti-oxidative and anti-cancer activities. The aim of this study was to investigate the protective effects of myricetin against B[a]P-induced toxicity. Treatment of B[a]P induced cytotoxicity on HepG2 cells, whereas co-treatment of myricetin with B[a]P reduced the formation of the B[a]P-7,8-dihydrodiol-9,10-epoxide (BPDE)-DNA adduct, which recovered cell viability. Furthermore, we found a protective effect of myricetin against B[a]P-induced genotoxicity in rats, via myricetin-induced inhibition of 8-hydroxy-2′-deoxyguanosine (8-OHdG) and BPDE-DNA adduct formation in the liver, kidney, colon, and stomach tissue. This inhibition was more prominent in the liver than in other tissues. Correspondingly, myricetin regulated the phase I and II enzymes that inhibit B[a]P metabolism and B[a]P metabolites conjugated with DNA by reducing and inducing CYP1A1 and glutathione S-transferase (GST) expression, respectively. Taken together, this showed that myricetin attenuated B[a]P-induced genotoxicity via regulation of phase I and II enzymes. Our results suggest that myricetin is anti-genotoxic, and prevents oxidative DNA damage and BPDE-DNA adduct formation via regulation of phase I and II enzymes.

## 1. Introduction

**Figure 1:**
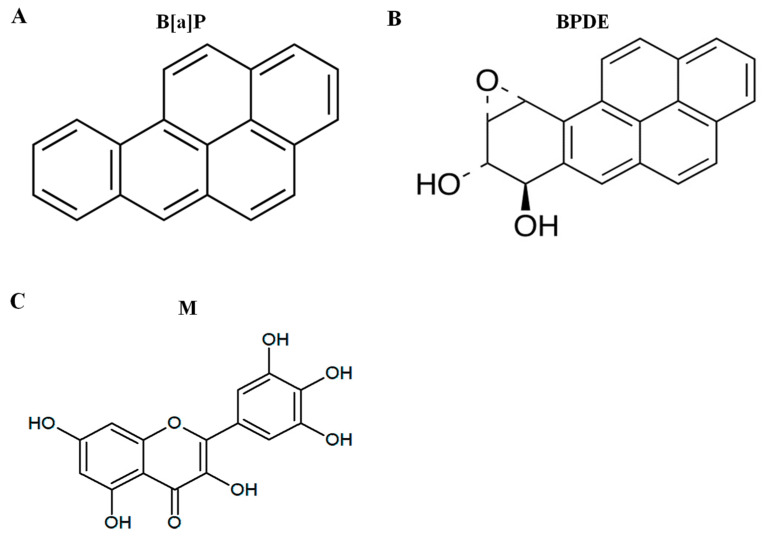
The structure of chemicals. (**A**) Benzo[a]pyrene (B[a]P), (**B**) B[a]P-7,8-dihydrodiol-9,10-epoxide (BPDE), and (**C**) myricetin.

Polycyclic aromatic hydrocarbons (PAHs) are ubiquitous environmental chemical carcinogens that lead to genetic damage and possess highly bioaccumulation characteristic [1]. Benzo[a]pyrene (B[a]P) is a well-known PAH (Figure 1A), which is listed as group 1 carcinogens by the International Agency for Research on Cancer (IARC). B[a]P is toxic and its exposure is primarily due to the food chain [2] Previous studies have shown that the average amount of people’s exposure to B[a]P is 8.09–9.20 ng/day [3]. This means that humans are exposed to a low dose of B[a]P over a lifetime. The long-term exposure of B[a]P can cause angiogenesis and metastasis in the skin, lungs, liver, colorectal and stomach [4]. Other studies support that long-term exposure of low-dose B[a]P induces cell angiogenesis and metastasis [5], and a low dose of B[a]P toxicity is enhanced by interaction with PM2.5 air pollutants [6]. B[a]P is converted to B[a]P-7,8-diol-9,10-epoxide (BPDE) which is ultimate metabolite of B[a]P (Figure 1B), and it leads to genetic toxicity via covalent binding with DNA [7]. In addition, B[a]P is linked to reactive oxygen species (ROS) formation, which induces genotoxicity via the formation of 8-hydroxy-2-deoxyguanosine (8-oxo-dG) [8].

B[a]P is metabolized by phase I enzymes, such as cytochrome P450 (CYP). Furthermore, CYP1A1 is associated with the B[a]P metabolism process [9]. Previous studies have shown that B[a]P is oxidized by CYP enzymes that induce a variety of B[a]P metabolite transitions that form DNA adducts [10,11]. After formation of DNA adducts, this leads to several diseases and cancers. The xenobiotic chemicals are generally converted into water-soluble metabolites by phase II enzymes in the cells, and are easily removed by phase III enzymes [12]. Uridine 5′-diphospho (UDP)-glucuronosyltransferases (UGTs), sulfotransferases (SULTs), glutathione S-transferases (GSTs), NAD(P)H: quinine oxidoreductase type 1 (NQO1), heme oxygenase-1 (HO-1), and *N*-acetyltransferase (NAT) are known as major phase II enzymes [13]. GST is a major enzyme of phase II detoxifying enzymes. It is activated when conjugated with BPDE and reduces DNA damage by counteracting B[a]P metabolites via inhibition of BPDE-DNA adduct formation [14,15]. In addition, GSH conjugates with B[a]P-7,8-dione, which attenuates the B[a]P-induced genotoxicity [16]. Following GSH–conjugation with B[a]P metabolites via phase II detoxifying enzymes, it is excreted by transporter genes, such as ABCC1 and ABCC2 [17]. Previous studies have showed that ABCC transporters are required to detoxify B[a]P [18]. The knockdown of ABCC2 increases the concentration of B[a]P metabolites in a variety of organs [19]. ABCC2 is required for the elimination of BPDE-DNA adducts in organs by B[a]P metabolite excretion.

B[a]P is produced during food processes such as broiling, frying, and roasting. These processes increase the concentration of B[a]P, which increases the risk of disease and cancer; therefore, it is important to prevent B[a]P-induced toxicity from the natural synthesis of B[a]P [20]. Natural compounds are widely used to reduce B[a]P-induced toxicity and in cancer therapy [21,22]. Myricetin (Figure 1C), which is found in vegetables, herbs, and fresh fruits, is used as a treatment against different cancers [23]. Previously, studies have showed that myricetin has potentially therapeutic properties, such as anti-oxidant, cytoprotective, and anti-cancer properties [24]. Additionally, another study has shown that myricetin is protective after B[a]P-induced DNA damage by regulating DNA strand breaks [25]. However, cancer-causing DNA mutations occur if the BPDE-DNA adducts and ROS-induced DNA damage are not repaired by base excision repair (BER) and nucleotide excision repair (NER). Therefore, it is important to prevent DNA damage by inhibiting DNA conjugation with B[a]P metabolites. In this study, we investigated the protective effect of myricetin against B[a]P-induced genotoxicity via the inhibition of DNA adduct formation by regulating phase I and II enzymes.

## 2. Materials and Methods

### 2.1. Chemicals and Reagents

Benzo[a]pyrene (B[a]P), myricetin, dimethyl sulfoxide (DMSO), ammonium persulfate, nuclease P1, N,N,N’N’-Tetramethyl ethylenediamine (TEMED), protease inhibitor cocktail, phosphatase inhibitor cocktail II, phosphatase inhibitor cocktail III, glycine, sodium dodecyl sulfate (SDS), tris base, sodium chloride, tween 20, and 2-mercaptoethanol were obtained from Sigma-Aldrich Chemical (St. Louis, MO, USA). Sodium pyruvate, penicillin-streptomycin, and trypsin-ethylenediaminetetraacetic acid (EDTA) were obtained from Welgene (Daegu, Korea). Alkaline phosphatase (AP) was purchased from Takara Bio Inc (Shiga, Japan). Phosphate-buffered saline (PBS) was purchased from Biosesang (Seongnam, Korea). A total of 30% acrylamide/bis solution was purchased from Bio-Rad (Hercules, CA, USA). Antibodies, such as CYP1A1, CYP1B1, GST, ABCC2, β-actin, and HRP-conjugated anti-rabbit immunoglobulin G (IgG), were purchased from Santa Cruz Biotechnology (Santa Cruz, CA, USA).

### 2.2. Cell Culture

Human-derived liver cancer cells (HepG2) were purchased from the American Type Culture Collection (Manassas, VA, USA). HepG2 cells were grown with Eagle’s minimum essential medium (MEM, Welgene, Daegu, Korea) containing 10% fetal bovine serum (FBS, Welgene), 100 μg/mL streptomycin, 100 U/mL penicillin, and 1 mM sodium pyruvate in 100 mm^2^ cell culture dishes. The old media were replaced with a new medium every two days. The HepG2 cells were incubated at 37 °C in 5% CO_2_ under a humidified atmosphere. The cells were used for further research when the confluency reached 80%.

### 2.3. Animals and Housing

Sprague-Dawley male rats (5-weeks-old; weight, 140–150 g) were purchased from Orient Bio (Seongnam-si, Korea) and were housed at a 23 ± 2 °C of temperature and 55 ± 1% of humidity. The room condition was maintained at specific pathogen free (SPF) with a 12 h light/12 h darkness cycle. Rodent chow and water were supplied ad libitum. The rats were randomly divided into three groups in each group (*n* = 6): (1) Control group, administration of corn oil (oral); (2) B[a]P-treated group, administration of B[a]P (2 mg/kg; daily, oral) dissolved in corn oil; (3) B[a]P co-treated with myricetin group, administration of myricetin (15 mg/kg) with B[a]P (2 mg/kg; daily, oral) dissolved in corn oil for 55 days. The concentration and period of B[a]P exposure were following other studies. To determine the B[a]P and myricetin concentration, we considered following reasons: (1) people are normally exposed to low-dose of B[a]P. Previous studies showed that people were exposed to 14 and 59.2 µg/kg/day of PAHs [26,27]. The average amount of people’s exposure to B[a]P is 8.09–9.20 ng/day [3]. Additionally, previous studies showed that short-term treatment of B[a]P for in vivo used the dose of B[a]P at 25 to 200 mg/kg in rats [28]. Moreover, another study suggested that low-dose of B[a]P concentration was defined under 12 mg/kg/day [29]. On the other hand, the animals were treated with doses of 50, 100, and 200 mg/kg of myricetin to rats for a long time [30]. (2) People are unavoidably exposed to B[a]P through foods and polluted air for a lifetime. Therefore, we consider the results and determine the long-term effects of a low dose of B[a]P and myricetin. Treatments were administered at the same time daily. Behavioral tests were performed in the morning. The animal experiment protocol was approved by Sungkyunkwan University Laboratory Animal Care Service (SKKU-2013-000105, 23 March 2013) in accordance with the Ministry of Food and Drug Safety (MFDS) Animal Protection of Korea (Oh-Song, Korea).

### 2.4. Cell Viability Assay

To evaluate the cytotoxicity of B[a]P and myricetin on HepG2 cells, a cell viability assay was performed. A density of 1 × 10^4^ cells/well of HepG2 cells were seeded in 96-well plates. A variety of concentrations of B[a]P (0, 1, 2.5, 5, and 10 µM) and myricetin (5, 10, 20, and 40 µM) were incubated in the wells for 48 h at 37 °C. After 48 h incubation, to evaluate the cell viability, 10 µL of EZ-CYTOX reagent (DOGEN, Daejeon, Korea) were treated with 100 µL MEM to each well and incubated for 2 h at 37 °C. Relative absorbance of each well was read at 450 nm to measure the amount of cell viability using a microplate reader (Molecular Devices, San Jose, CA, USA).

### 2.5. BPDE-DNA Adduct Formation Analysis

DNA extraction was performed using a QIAamp DNA Mini Kit (Qiagen, Valencia, CA, USA), according to the manufacturer’s instructions. DNA was isolated and the level of BPDE-DNA adduct formation was assessed using BPDE-DNA adduct ELISA kit (Cell Biolabs, San Diego, CA, USA) and following manufacturer’s instructions. Briefly, DNA samples of 2 µg/mL concentration are prepared. An amount of 100 µL of each sample is treated in 96 well plates for 2 h at 37 °C and rinsed two times. After washing, 100 µL of anti-BPDE antibody is treated in each well and incubated for 2 h at room temperature. Each well is rinsed 5 times using washing buffer, and then secondary antibody is added in each well for 1 h at room temperature. Each well is rinsed 3 times using washing buffer. All wells are reacted with 3,3′,5,5′-Tetramethylbenzidine (TMB) buffer at room temperature for 20 min. Finally, the reaction is stopped by adding stop solution. The BPDE-DNA adducts formation level was evaluated by measuring the relative absorbance using a microplate reader at 450 nm.

### 2.6. Quantification of DNA Damage via 8-Hydroxydeoxyguanosine

We evaluated the concentration of 8-hydroxydeoxyguanosine (8-OHdG) using an 8-OHdG DNA damage ELISA kit (Cell Biolabs, San Diego, CA, USA), according to manufacturer’s instructions. Briefly, isolated DNA was denatured at 95 °C for 5 min and immediately transferred to ice. A total of 10 units of nuclease P1 and 20 mM sodium acetate (pH 5.2) were added to total DNA, and digested DNA to nucleosides for 2 h at 37 °C. Next, alkaline phosphatase (Takara, Japan) added for 15 min at 37 °C, followed by incubation for 15 min at 50 °C in 100 mM Tris buffer (pH 7.5). The reaction mixtures were centrifuged for 5 min at 6000× *g* and the supernatants were extracted for assay analysis. Briefly, 50 µL of each sample is treated in wells and incubated for 10 min at room temperature. An amount of 50 µL of anti-8-OHdG antibody is treated in each well and incubated for 1 h at room temperature on orbital shaker. Each well is rinsed 3 times, 100 µL secondary antibody is added in each well and incubated for 1 h at room temperature. After incubation, 100 µl of substrate solution buffer is added in each well for 20 min at room temperature and then the reaction is stopped by adding stop solution. The absorbance of 8-OHdG was measured using a microplate reader at 450 nm (VERSA max™, Molecular Devices, San Jose, CA, USA).

### 2.7. Western Blot Analysis

The protein in the liver was extracted using PRO-PREP^™^ protein extraction solution (iNtRON, Seongnam, Korea). The protein in cells was extracted in RIPA buffer (150 mM NaCl, 1% Nonidet P-40, 50 mM Tris-HCl, and 0.25% sodium deoxycholate) (Biosolution, Seoul, Korea) containing protease inhibitor cocktail, phosphatase inhibitor cocktail II and III. Each protein concentration was quantified using the Bio-Rad protein assay (Hercules, CA, USA). After protein quantification, it was denatured at 95 °C for 5 min in buffer. Next, samples (50 µg) were ran on 10% SDS-polyacrylamide gel electrophoresis (SDS-PAGE) at 50 V for 60 min in running buffer. The proteins were transferred to polyvinylidene difluoride (PVDF) membranes (BioRad, Hercules, CA, USA) at 100 V for 90 min in a transfer buffer. Membranes blocked with TNT buffer containing 5% skim milk for 1 h, followed by incubation with CYP1A1, CYP1B1, and β-actin antibodies overnight at 4 °C. After washing for 15 min at 4 times with TNT buffer, the membrane was incubated for 45 min with secondary antibodies, and then washed for 15 min at 4 times with TNT buffer. Immunoreactivity was visualized using chemiluminescence (ECL) Plus Western Blotting reagents (Amersham Bioscience, Buckinghamshire, UK). The protein level was quantified using Quantity One Image Software (Bio-Rad, Hercules, CA, USA).

### 2.8. Statistical Analysis

Experimental data were evaluated in triplicates and experiments were repeated at least three times. All data were expressed as mean ± standard error of the mean (SEM). The One-way analysis of variance (ANOVA) and Tukey’s multiple comparison analysis were performed to determine the significant differences between groups using GraphPad Prism 5.0 (GraphPad Software, San Diego, CA, USA). Differences were considered statistically significant when *p* < 0.05.

## 3. Results

### 3.1. Protective Effect of Myricetin against B[a]P-Induced Toxicity

**Figure 2:**
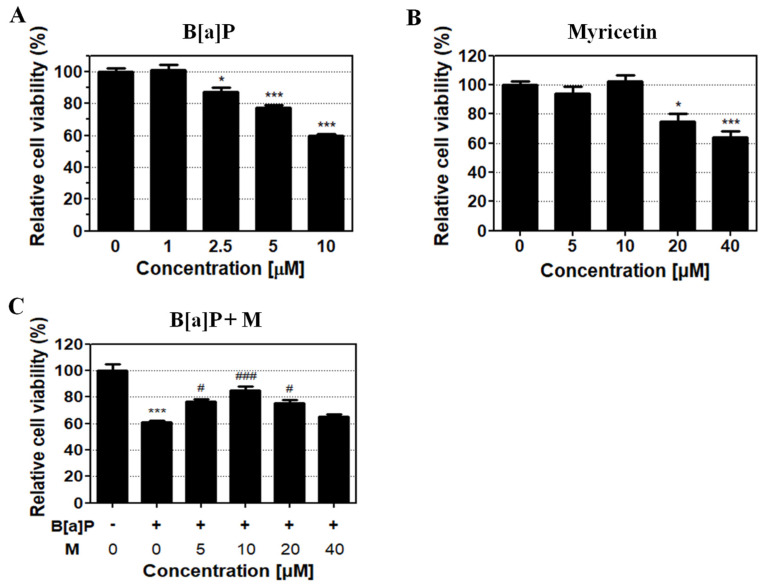
B[a]P and myricetin cytotoxicity in HepG2 cells. (**A**,**B**) HepG2 cells were treated with B[a]P (0, 1, 2.5, 5, and 10 µM) or myricetin (0, 5, 10, 20, and 40 µM) at different concentrations for 48 h. (**C**) The protective effect of myricetin against B[a]P-induced cytotoxicity was measured with B[a]P (10 μM) co-treatment with various concentrations of myricetin for 48 h. All treatment group values are significantly different when compared with controls (* *p* < 0.05, *** *p* < 0.001) and B[a]P (# *p* < 0.05, ### *p* < 0.001). Tukey’s multiple comparison test. M: myricetin.

HepG2 cells were treated with different concentrations of B[a]P and myricetin to evaluate their toxicity. B[a]P treatment for 48 h induced cytotoxicity in a dose-dependent manner compared with the control group (Figure 2A). The 40% inhibitory concentration of B[a]P was calculated at 10 µM for 48 h and used in subsequent experiments. Treatment of myricetin at 5, 10, 20, and 40 µM for 48 h revealed no toxicity up to 10 µM; however, cytotoxicity was shown for treatments > 20 µM when compared with the control group (Figure 2B). Then, to confirm the protective effect of myricetin on B[a]P cytotoxicity, HepG2 cells were co-treated with or without B[a]P in the presence of myricetin and the amount of the viable cells continuously analyzed compared with non-treated group. We found that myricetin co-treatment with B[a]P recovered up to 85% cell viability (Figure 2C). These results suggest that myricetin has protective effect against B[a]P-induced cytotoxicity. Based on the cell viability data, 10 µM of myricetin was used for all further in vitro experiments.

### 3.2. Protective Effects of Myricetin against B[a]P-Induced Oxidative DNA Damage

**Figure 3:**
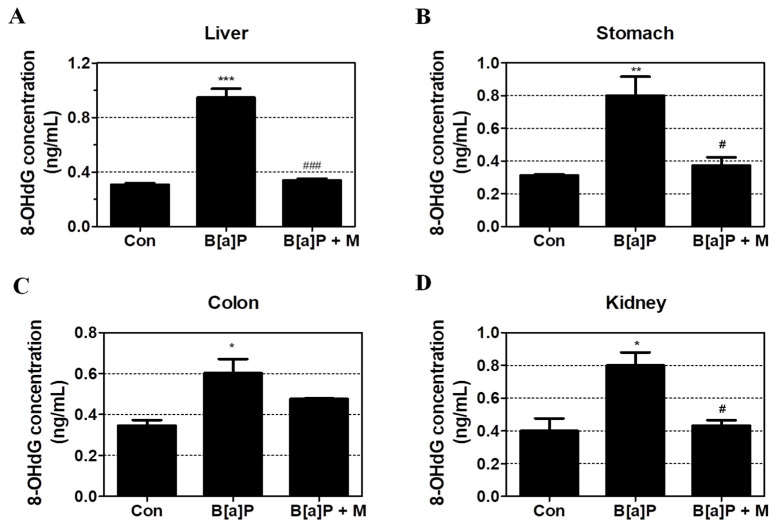
Oxidative DNA damage following B[a]P (2 mg/kg) and myricetin (10 mg/kg) co-treatment. Oxidative DNA damage was measured by the concentration of 8-OHdG in liver, stomach, colon, and kidney tissues. (**A**–**D**) Myricetin was protective against B[a]P-induced 8-OHdG formation following B[a]P treatment in all tissue when compared with controls (* *p* < 0.05, ** *p* < 0.01, *** *p* < 0.001) and B[a]P (# *p* < 0.05, ### *p* < 0.001). Tukey’s multiple comparison test. M: myricetin.

To confirm the B[a]P-induced oxidative DNA damage, we calculated the concentration of 8-OHdG in the liver, kidney, colon, and stomach tissue of rats following treatment with 2 mg/kg of B[a]P and 15 mg/kg of myricetin for 55 days. B[a]P significantly increased the concentration of 8-OHdG in the liver, stomach, colon, and kidney when compared with the control group but B[a]P co-treatment with myricetin significantly reduced the 8-OHdG in the liver, stomach, and kidney respectively (Figure 3A–D). These results confirmed that myricetin protects DNA from further oxidation that contributes to cell death prevention.

### 3.3. Inhibition Effect of Myricetin against BPDE-DNA Adduct Formation

**Figure 4:**
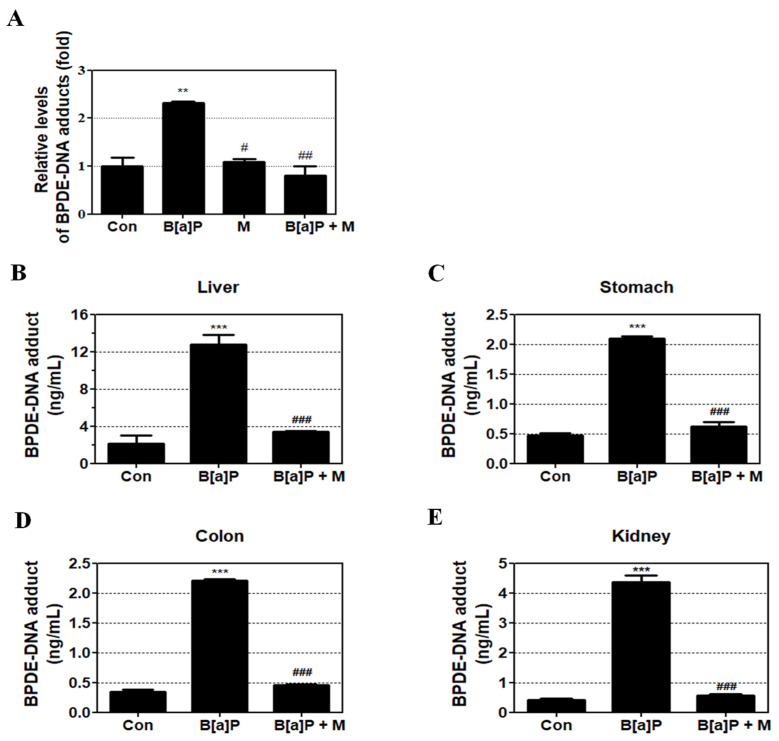
BPDE-DNA adduct concentration in B[a]P and B[a]P + myricetin treated groups. (**A**) Effect of myricetin on BPDE-DNA adduct formation in HepG2 cells following treatment with B[a]P (10 μM) and co-treatment with myricetin (10 μM) for 48 h. (**B**–**E**) In Sprague-Dawley rats, treatment with B[a]P (2 mg/kg) and co-treatment with myricetin (15 mg/kg) for 55 days. All treatment groups are significantly different when compared with controls (** *p* < 0.01, *** *p* < 0.001) and B[a]P (# *p* < 0.05, ## *p* < 0.01, ### *p* < 0.001). Tukey’s multiple comparison test. M: myricetin.

To evaluate the protective effect of myricetin against B[a]P-induced genotoxicity, the concentration of BPDE-DNA adducts was measured in HepG2 cells and the liver, stomach, colon, and kidney tissue of rats using BPDE-DNA Adduct ELISA Kit. Treatment with B[a]P, myricetin, and B[a]P + myricetin showed that B[a]P induced BPDE-DNA adduct formation when compared with the non-treatment group. However, B[a]P co-treatment with myricetin clearly decreased BPDE-DNA adduct formation when compared with B[a]P treatment alone (Figure 4A). Furthermore, B[a]P induced BPDE-DNA adduct formation in the liver, kidney, colon, and stomach of treated rats when compared with the control group. B[a]P co-treatment with myricetin inhibited the BPDE-DNA adduct formation in all rat tissue (Figure 4B–E). These results showed that the genotoxicity of B[a]P was attenuated by myricetin via inhibition of the formation of BPDE-DNA adduct.

### 3.4. Regulatory Effect of Myricetin on the Expression of Phase I, II, and III Enzyme

**Figure 5:**
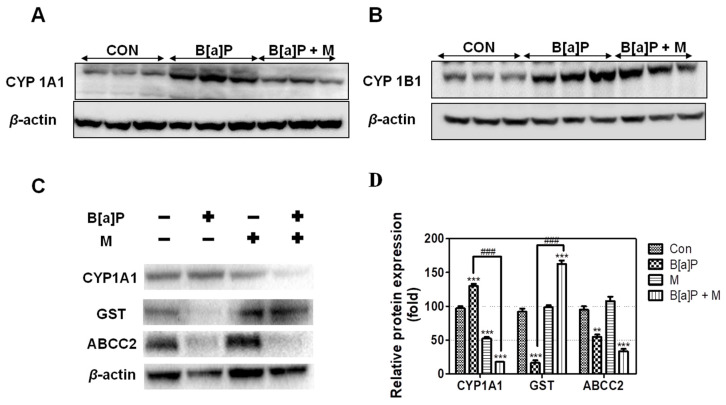
The expression of phase detoxifying enzymes. (**A**,**B**) The effect of myricetin (15 mg/kg) and B[a]P (2 mg/kg) treatment on CYP1A1 and CYP1B1 enzyme expression in the liver of Sprague-Dawley rats. (**C**) The effect of myricetin (10 μM) and B[a]P (10 μM) treatment on CYP1A1, GST, and ABCC2 enzyme expression in HepG2 cells. (**D**) Quantitative evaluation of relative protein expression of CYP1A1, GST, and ABCC2. All treatment groups are significantly different when compared with controls (** *p* < 0.01, *** *p* < 0.001) and B[a]P (### *p* < 0.001). Tukey’s multiple comparison test. M: myricetin.

Our results showed that B[a]P induced CYP1A1 and CYP1B1 expression. In contrast, B[a]P + myricetin co-treatment down-regulated CYP1A1, but not CYP1B1, expression in the liver of rats (Figure 5A,B). We confirmed that myricetin co-treatment with B[a]P reduced CYP1A1 expression when compared with B[a]P treatment group (Figure 5C,D). This indicated that myricetin regulated CYP1A1 expression, thereby reducing B[a]P metabolism, which prevented DNA adduct formation.

Next, we measured GST expression in HepG2 cells following B[a]P and B[a]P + myricetin treatment. B[a]P reduced GST expression; however, co-treatment with myricetin recovered this expression when compared with B[a]P treatment alone (Figure 5C,D). In contrast, ABCC2 was not regulated by myricetin. This indicated that myricetin attenuated B[a]P-induced toxicity via reduction of oxidative DNA damage and inhibition of BPDE-DNA adduct formation by inducing GSH conjugated with B[a]P metabolites to recover the GST expression.

## 4. Discussion

B[a]P is carcinogenic, and is formed during food-processing, and in tobacco smoke, waste products, and industry [31,32]. B[a]P is well-known as an ubiquitous environmental agent. Previous studies showed that people were exposed to 8.09–9.20 ng/day of B[a]P [3]. This report indicates that people are exposed to a low dose of B[a]P over their lifetime. Generally, B[a]P accumulated in humans through food; 97% of B[a]P exposure amount is associated with the food chain [2]. This indicates that the exposure of B[a]P is mainly mediated in the gastrointestinal tract such as stomach and colon. Absorbed B[a]P through the gastrointestinal tract passes through the blood vessel to the liver and undergoes the detoxification process. Then, detoxified metabolites are excreted via the urine or bile. Thus, in this study, the liver, kidney, and gastrointestinal tract (stomach and colon) were considered as target organs. After exposure to B[a]P, it is metabolized to BPDE or B[a]P radical cations, which are conjugated with DNA. Previous studies have shown that B[a]P induces genotoxicity by forming BPDE-DNA adducts or 8-OHdG [33]. Additionally, another study shows that the long-term treatment of low-dose B[a]P induces the cancer progression [5]. This means that B[a]P negatively affects the people’s health causing dysfunction of hormone, cancers, and autoimmune disease [34]. Previous study shows that inhibition of DNA adduct formation reduces B[a]P mutagenesis and carcinogenesis [35]. Many studies have assessed the effect of flavonoids in reducing the B[a]P-induced toxicity [36,37,38].

Myricetin, which is present in many foods such as tea, vegetables, and medical plants, is a natural flavonoid that has anti-carcinogenic, -oxidant, and -inflammation effects [39,40]. Generally, the protective effects of myricetin against oxidative stress have been well-studied for a long time. Previous studies have shown that the anti-oxidant effect of myricetin is mediated by its ROS scavenging activity and activation of cellular anti-oxidative mechanisms including nuclear factor erythroid 2-related factor 2 (Nrf2)-linked pathway [41,42,43]. Myricetin is protective against B[a]P-induced DNA damage via oxidized pyrimidine. In contrast, oxidized purines are not reduced by myricetin [25]. This means that myricetin does not repair after receiving purine-based damage by B[a]P. Therefore, the inhibition of 8-OHdG and BPDE-DNA adducts is important for the attenuation of B[a]P-induced DNA damage. In this study, we provide new insights into the protective effects of myricetin against B[a]P-induced toxicity.

We confirmed that myricetin recovered cell viability in HepG2 cells, indicating that myricetin could attenuate B[a]P-induced cytotoxicity (Figure 2C). Previous studies have shown that the formation of DNA adducts induces B[a]P genotoxicity via oxidative stress and BPDE. B[a]P is transited to B[a]P-7,8-dione, which enhances ROS production by inducing futile redox cycles [44]. The formation of 8-OHdG is an oxidative DNA damage marker [45]. In addition, B[a]P is sequentially transited to B[a]P-7,8-epoxide, B[a]P-7,8-dihydrodiol, and BPDE, which is a metabolite of B[a]P that causes genotoxicity via the formation of BPDE-DNA adducts [35]. On the other hand, the amount of oxidative DNA damage and the level of BPDE formation are different depending on the types of cell lines and organs [46]. Thus, to evaluate the effect of myricetin on B[a]P-induced toxicity, we analyzed 8-OHdG and BPDE-DNA adducts using HepG2 cells and various organs such as the liver, kidney and gastrointestinal tract (stomach and colon). Our results confirmed that B[a]P induced 8-OHdG and BPDE-DNA adduct formation, respectively. In contrast, co-treatment with myricetin and B[a]P reduced 8-OHdG and BPDE-DNA adduct formation in all organ tissues (Figure 3 and Figure 4). The results indicate that reduction of B[a]P-induced cytotoxicity is induced by myricetin through reducing B[a]P metabolites-DNA adducts formation. To examine whether the side-effects affect the animals by treatment with B[a]P or myricetin, we evaluated the effect of B[a]P and myricetin on body and organ weights. We confirmed that the weights of organs including the liver, stomach, colon, and kidney were not changed in all treatment groups (supplementary information, Figure S1B–E). Moreover, the bodyweight of animals was not significantly different between the treated groups and untreated control groups (supplementary information, Figure S1A). These results indicated that there were no significant side-effects during long-term exposure to B[a]P and myricetin. Therefore, our results show that myricetin attenuates B[a]P-induced genotoxicity mainly by inhibiting oxidative DNA damage and BPDE-DNA adduct formation.

B[a]P-mediated genotoxicity is caused by the interaction of B[a]P metabolites with DNA. Our results show that myricetin reduces B[a]P-induced genotoxicity by reducing the formation of B[a]P metabolites-DNA adduct. We hypothesize that myricetin inhibits the opportunity of B[a]P metabolites conjugated with DNA. Attenuation of B[a]P-DNA adduct formation was considered to be mediated by two factors: (1) reduction in conversion of B[a]P to B[a]P metabolites, and (2) elimination of B[a]P metabolites. Previous studies have showed that the conversion of B[a]P to BPDE requires multi-enzymatic steps [47]. B[a]P is metabolized by CYP 450; its metabolites cause 8-OHdG and BPDE-DNA adduct formation. A report shows that B[a]P metabolism is regulated by CYP1A1 and CYP1B1, which are associated with B[a]P-mediated carcinogenesis [48]. CYP 450 enzymes induce B[a]P conversion to its metabolites, which conjugate with DNA to form carcinogenic DNA adducts [49]. However, our results showed that CYP1A1, but not CYP1B1, expression was decreased by myricetin treatment when compared with B[a]P alone (Figure 5A,B). A previous study has reported that the regulation of BPDE-DNA adduct formation is correlated with CYP1A1 [50]. Furthermore, the attenuation of cellular DNA damage in HepG2 cells is associated with a reduction in oxidative stress via suppression of CYP1A1 [51]. Indeed, CYP1A1 is the most efficient enzyme forming intermediate metabolite of B[a]P-derived DNA adducts in the liver [52]. Taken together with our data, this indicates that myricetin-induced attenuation of B[a]P genotoxicity is caused by reducing the intermediate metabolites of B[a]P-derived DNA adducts via regulation of CYP1A1 expression.

GST is a phase II enzyme that is conjugated with xenobiotics, including B[a]P, which is a well-known detoxification system in the body [53]. One previous study has shown that GST contributes to a reduction in BPDE-DNA adduct formation and 8-OHdG by inducing GSH-conjugation with B[a]P metabolites [54]. Our results showed that B[a]P reduced GST expression, whereas myricetin recovered this effect (Figure 5C,D). These results suggested that myricetin attenuates B[a]P-induced DNA damage by inhibiting 8-OHdG and BPDE-DNA adduct formation via GST expression induction. In contrast, phase III enzymes are not significantly regulated by myricetin (Figure 5C,D). We hypothesize that myricetin regulates other transporter genes or does not affect phase III enzymes. Previous studies have shown that myricetin induces anticancer drug efficiency by modulating drug efflux via transporter gene regulation [55,56]. In addition, many transporter genes have been shown to localize to the basolateral or canalicular membrane, which is associated with the excretion of drugs through the bile or blood [57]. Previous studies have revealed that GSH interacting with BPDE enhances the BPDE solubility in water, which is eliminated by ABCCs [58]. Therefore, further studies are required to assess the excretion mechanism of B[a]P metabolites that are conjugated with phase II enzymes by myricetin. Taken together, myricetin attenuates B[a]P-induced 8-OHdG and BPDE-DNA adduct formation by regulating CYP1A1 and GST expression.

## 5. Conclusions

This study shows that myricetin reduces B[a]P-induced toxicity by inhibiting BPDE-DNA adduct and 8-OHdG formation. The inhibition of B[a]P-induced genotoxicity is associated with two factors: (1) a reduction of the B[a]P metabolism via reduced CYP1A1 expression, and (2) the elimination of B[a]P metabolites via enhanced GST expression. These results indicate that myricetin inhibits both B[a]P conversion to B[a]P metabolites and B[a]P metabolites conjugated with DNA, thereby inhibiting the formation of 8-OHdG and BPDE-DNA adducts. Our study suggests that myricetin is protective against B[a]P-induced genotoxicity by inhibiting oxidative DNA damage and BPDE-DNA adduct formation.

## Supplementary Materials

The following are available online at https://www.mdpi.com/2076-3921/9/5/446/s1, Figure S1: Effect of B[a]P and myricetin on body and organ weights. B[a]P (2 mg/kg) alone or together with myricetin (15 mg/kg) was administered to Sprague-Dawley rats orally for 55 days. (**A**) Body weight changes of the rats during 55 days. (**B–E**) Organ weights of the rats after 55 days. M: myricetin.
