# Supplementary material for: Protective Effects of Myricetin on Benzo[a]pyrene-Induced 8-Hydroxy-2′-Deoxyguanosine and BPDE-DNA Adduct
Source: Antioxidants (Basel). 2020 May 21;9(5):446. doi: 10.3390/antiox9050446 (PMC7278665; doi:10.3390/antiox9050446)
Supplement: Supplementary file 1 [file antioxidants-09-00446-s001.pdf]

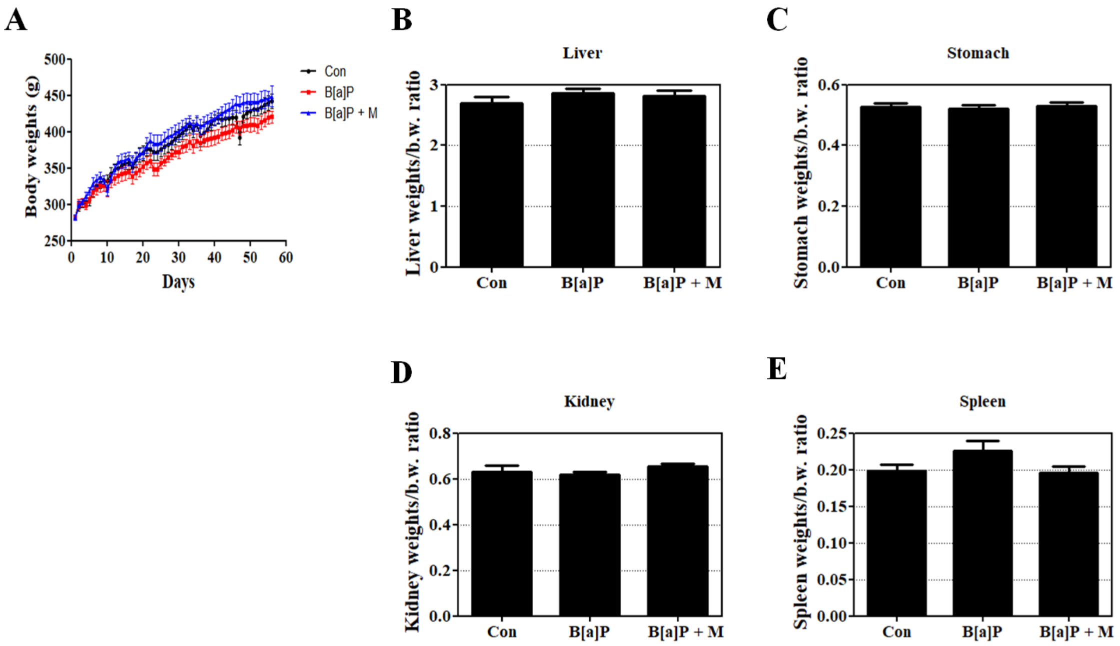

Figure S1. Effect of B[a]P and myricetin on body and organ weights. B[a]P (2 mg/kg) alone or together with myricetin (15 mg/kg) was administered to Sprague-Dawley rats orally for 55 days. (**A**) Body weight changes of the rats during 55 days. (**B–E**) Organ weights of the rats after 55 days. M: myricetin.
